# Laboratory predictors for risk of revision surgery in pediatric septic arthritis

**DOI:** 10.1007/s11832-016-0736-6

**Published:** 2016-05-12

**Authors:** Jessica J. M. Telleria, Rosemary A. Cotter, Viviana Bompadre, Suzanne E. Steinman

**Affiliations:** Department of Orthopaedics and Sports Medicine, University of Washington, 1959 NE Pacific St., Seattle, WA 98195 USA; Department of Orthopaedics and Sports Medicine, OA.9.120.1—Orthopedics Administration, Seattle Children’s Hospital, 4800 Sand Point Way NE, Seattle, WA 98105 USA

**Keywords:** Pediatric, Septic arthritis, Surgery, Failure, Blood culture, C-reactive protein

## Abstract

**Background:**

Reported complications of pediatric septic arthritis range from minor growth abnormalities to potentially life-threatening conditions and death; some children require multiple surgeries for eradication of infection. The purpose of this study is: (1) to determine the failure rate of a single surgical incision and drainage (I&D) in pediatric septic arthritis, (2) to identify risk factors for failure which are detectable at the time of initial presentation, and (3) to trend post-operative C-reactive protein (CRP) values to see if there is a difference between children who fail a single I&D and those who do not.

**Methods:**

The medical records for 105 children who underwent operative management of septic arthritis were retrospectively reviewed. Single and multivariate analyses were performed.

**Results:**

Eighty-four children required one surgical intervention [mean age 5.18 years (±4.01); 38 females (45 %), 46 males (55 %)], 21 children required revision surgery [mean age 8.16 years (±4.54); 4 females (19 %), 17 males (81 %)], and the overall rate of revision surgery was 20 %. Delayed diagnosis (*p* = 0.015), elevated CRP at presentation (*p* = 0.000), positive blood culture (*p* = 0.000), and age (*p* = 0.009) were all associated with revision surgery in bivariate analysis. In multivariate analysis, CRP at presentation and positive blood culture were significant risk factors for revision surgery (*p* = 0.005 and *p* = 0.025, respectively). Additionally, markedly elevated CRP levels on post-operative days (POD) 1–4 were each independently significant risk factors for requiring multiple surgeries (all *p* < 0.000). Fever, elevated erythrocyte sedimentation rate, and leukocyte count were not risk factors for multiple surgeries.

**Conclusions:**

In this study, a positive blood culture or marked elevation in CRP at presentation or on POD 1–4 were associated with revision surgery. These findings may help improve surgical planning for both the initial surgery in order to avoid revisions, as well as revision surgery, should it be indicated.

**Level of evidence:**

III.

**Electronic supplementary material:**

The online version of this article (doi:10.1007/s11832-016-0736-6) contains supplementary material, which is available to authorized users.

## Introduction

Septic arthritis is an emergency comprising 21 % of acute pediatric musculoskeletal infections [1], with a reported incidence of 5–12 cases per 100,000 person-years [2]. The associated morbidity and mortality has diminished significantly in the modern era, but serious sequela such as growth disturbance, arthritic changes, avascular necrosis, pathologic fracture, septic thrombophlebitis, and death are still reported in the setting of severe musculoskeletal infection [1, 3–10]. With few exceptions, once identified, these patients undergo incision and drainage (I&D) of the affected joint and begin intravenous antibiotics. The majority of children improve after a single I&D and a 3–6-week course of antibiotics. However, similar to adult populations [11–13], a subset of children require multiple operative procedures for resolution of infection.

Serum laboratory studies, including C-reactive protein (CRP), erythrocyte sedimentation rate (ESR), and leukocyte count (WBC), are ubiquitously obtained as initial screening measures and are mainstays in the diagnosis of septic arthritis in children [3, 4, 14–18], especially in centers where urgent advanced imaging is not readily available. Serum inflammatory marker kinetics in uncomplicated pediatric septic arthritis and osteomyelitis have previously been reported [4, 14, 15, 19], and laboratory predictors for long-term sequela in acute pediatric osteomyelitis have been described [4]. Furthermore, risk factors for failed surgical I&D of septic arthritis in adult populations has recently been reported [13]. However, in our review of the English-language literature, no study to date has sought to explore objective differences between children with septic arthritis who failed initial surgical I&D, necessitating additional operative intervention, and children who experienced rapid clinical improvement. This information would be of clinical value in identifying at-risk children early during their disease course, so as to better improve patient care, minimize complications, and facilitate initial family counseling.

Therefore, the purpose of this study is to: (1) determine the failure rate of a single surgical intervention in pediatric septic arthritis, (2) to identify risk factors for failure of a single intervention which are detectable at the time of initial presentation, and (3) to trend post-operative CRP values to see if there is a difference between children who fail a single I&D and those who do not.

## Materials and methods

### Patient population

All patients who underwent surgical management of septic arthritis between January 1, 2006 and September 1, 2013 were identified through a retrospective search of a single-hospital orthopedic database. Institutional Review Board approval was obtained.

Inclusion criteria were children less than 18 years of age with confirmed septic arthritis of the shoulder, elbow, wrist, hip, knee, or ankle. Exclusion criteria included patients with post-operative wound infections, necrotizing soft tissue infection (NSTI), septic arthritis associated with osteomyelitis, and patients with inflammatory, rheumatologic, or immunosuppressive conditions. Patients who transferred into, or out of, the investigative institution during their inpatient course were also excluded.

### Operative management and hospital course

Children who presented with a history concerning for septic arthritis received a comprehensive history and physical, laboratory, and radiographic evaluation. Normal laboratory references were CRP less then 0.8 mg/dL, ESR 0–10 mm/h, and WBC 5000–11,000 cells/mm^3^. Routine radiographs were obtained; when indicated, advanced imaging or ultrasound was performed. Presumed septic arthritis was diagnosed using established criteria [16, 17, 20] and confirmed by (1) positive synovial fluid Gram stain and/or culture [20, 21], (2) frank purulence expressed from the joint at surgery [21–23], or (3) aspirated synovial fluid nucleated cell count greater than 50 × 10^3^ cells/L [20, 21].

Routine open or arthroscopic incision and drainage was performed (Table 1). All joints were lavaged with a high volume of sterile saline, with or without added antibiotic. When necessary, partial capsulectomy, synovectomy, cortical windowing, and intramedullary drilling were performed. Typically, the capsule was left loosely approximated to allow for additional drainage, and a surgical drain was placed in all cases (Table 1). Surgery was performed by one of ten pediatric orthopedists. Post-operatively, patients received either an intravenous first-generation cephalosporin, clindamycin for penicillin allergy, or vancomycin for a history of methicillin-resistant *Staphylococcus aureus* (MRSA). Infectious disease consultation was obtained per protocol and antibiotics were tailored appropriately as culture results became available. Laboratory parameters were trended per the following protocol: CRP every other day, WBC every 2–4 days, and ESR weekly. Discharge criteria included obvious clinical improvement, resolution of fever and CRP <2.0 mg/dL; occasionally, children with marked clinical improvement and a strongly down-trending CRP were discharged before CRP passed below 2.0 mg/dL. Per protocol, patients continued intravenous antibiotics until discharge, and then switched to an appropriate oral antibiotic as determined by the infectious disease team.

**Table 1:** Surgical intervention details

Intervention	Single surgery, *n* (%)	Multiple surgeries, *n* (%)	Total, *n* (%)
Number of patients	84	21	105
Procedure			
Open	77 (91.7 %)	20 (95.2 %)	97 (92.4 %)
Arthroscopic	7 (8.3 %)	1 (4.8 %)	8 (7.6 %)
High-volume lavage	84 (100 %)	21 (100 %)	105 (100 %)
Antibiotic irrigant	29 (34.5 %)	8 (38.1 %)	37 (35.2 %)
Capsulectomy			
Complete	0 (0 %)	0 (0 %)	0 (0 %)
Partial	28 (33.3 %)	9 (42.9 %)	37 (35.2 %)
Synovectomy	0 (0 %)	1 (4.8 %)	1 (1.0 %)
Cortical window/bone drilled	11 (13.1 %)	4 (19.0 %)	15 (14.3 %)
Antibiotic PMMA beads	0 (0 %)	0 (0 %)	0 (0 %)
Capsular closure	20 (23.8 %)	4 (19.0 %)	24 (22.9 %)
Drain placed	84 (100 %)	21 (100 %)	105 (100 %)

Technical details of the initial surgical procedure performed on children with septic arthritis. Eighty-four patients underwent a single surgery and 21 required multiple surgeries

Children who failed to demonstrate rapid clinical improvement post-operatively were monitored carefully. Patients who required revision surgery were identified by clinical deterioration, continued fever, persistently elevated inflammatory laboratory values, erythematous, and/or purulent draining wounds.

### Statistical methods

All pre- and post-operative patient records, operative notes, radiologic studies, and laboratory results were reviewed. Study data were collected and managed using the Research Electronic Data Capture (REDCap) tool (Vanderbilt University, Nashville, TN) [24].

Differences between groups were estimated using Wilcoxon rank-sum tests for continuous variables and the Chi-square test for categorical variables. Stepwise variable selection using logistic regression analysis was performed to identify risk factors for revision surgery. The model included variables with a *p*-value <20. CRP trends were analyzed using repeated measures analysis of variance (ANOVA) with Bonferroni post-hoc pairwise comparison. A receiver operating characteristic (ROC) curve was constructed to determine the diagnostic performance for identifying patients who would require multiple surgeries. Statistical significance was set at *p* ≤ 0.05 and all confidence intervals at 95 %.

### Source of funding

Funding provided by an internal departmental grant, no external funding sources. The database management system utilized in this study (REDCap) has received grant support from NCRR/NIH (UL1 RR025014) [24].

## Results

Over the study period, 168 continuous patients with presumed septic arthritis were identified. The following children were excluded: 12 for post-operative infection, four for superficial extra-articular infection, two for isolated osteomyelitis and 18 for osteomyelitis associated with septic arthritis as documented on magnetic resonance imaging (MRI), one for NSTI, 14 for inflammatory/rheumatologic condition, seven for immunosuppression, and five patients were transferred during their inpatient course. The final study population comprised 105 patients with confirmed septic arthritis; 84 (80 %) children required one incision and drainage and 21 (20 %) required multiple surgeries (Table 2). The mean age of the single and multiple surgery cohorts were 5.18 years (range 0.3–15.9 years) and 8.16 years (range 1.3–17.5 years), respectively; no newborns were included in the study. The hip was the most commonly affected joint (48.6 %), followed by knee (31.4 %), ankle (8.6 %), elbow (4.8 %), and shoulder (2.9 %). There was one infected wrist, one subtalar joint, one patient who presented with both an infected shoulder and hip, and one patient with bilateral septic hips.

**Table 2:** Bivariate analysis of key features at presentation

	Single surgery		Multiple surgeries		*p*-Value
	Mean (SD), *n* = 84	Median (IQR)	Mean (SD), *n* = 21	Median (IQR)	
Age (years)	5.18 (±4.01)	4.18 (6.27)	8.16 (±4.54)	7.17 (6.25)	0.009*
Gender (F)	38 (45 %)		4 (19 %)		0.028*
Days antecedent malaise	4.04 (±3.73)	3 (5)	5.76 (±4.1)	5 (6)	0.118
Delayed Dx^a^	35 (42 %)		15 (71 %)		0.015*
Inability to WB^a^	76 (93 %)		18 (90 %)		0.689
Prior antibiotics^a^	16 (19 %)		7 (33 %)		0.157
Temp (°C)	37.98 (±1.08)	37.7 (1.4)	38.1 (±1.05)	38 (0.79)	0.677
WBC (10^3^cell/mm^3^)	12.94 (±4.52)	12.1 (6.5)	13.13 (±7.05)	13 (12.6)	0.926
ANC (cell/mm^3^)	7516 (±3172)	6771 (3570)	9110 (±5631)	8016 (8902)	0.673
ESR (mm/h)	47.95 (±29.5)	40 (43)	52.16 (±30.32)	51 (53)	0.591
CRP at presentation (mg/dL)	7.91 (±7.14)	4.7 (7.5)	17.64 (±9.85)	15.85 (13.12)	0.000*
Positive blood Cx^a^	19 (24 %)		14 (66 %)		0.000*

Bivariate analysis of key clinical and diagnostic factors at presentation in children with septic arthritis who required either a single or multiple surgical debridements for resolution of infection

*SD* standard deviation; *IQR* interquartile range; *F* female; *Dx* diagnosis; *WB* weight bear; *CRP* C-reactive protein; *Cx* culture

* *p* ≤ 0.05

^a^Frequencies (percentage)

Clinical and laboratory parameters between study groups at the time of initial presentation are shown in Table 2. Bivariate analysis revealed that CRP at presentation (*z* = −4.15; *p* = 0.000), positive blood culture (χ^2^ = 13.92; *p* = 0.000), increasing age (*z* = −2.59; *p* = 0.009), male sex (χ^2^ = 4.8; *p* = 0.028), and delayed diagnosis (χ^2^ = 5.97; *p* = 0.015) were all associated with multiple surgeries. Children with a delay in diagnosis were initially seen by another provider and thought to have a benign condition, most commonly viral upper respiratory tract infections, toxic synovitis, or minor sprains or contusions.

Multivariate analyses showed that CRP at presentation [odds ratio (OR) = 1.1; 95 % confidence interval (CI) = 1.03–1.18; *p* = 0.005] and having a positive blood culture (OR = 3.9; 95 % CI = 1.18–12.8; *p* = 0.025) were predictors for revision surgery (Table 3). That is, for every one-unit of increase in CRP, the odds of undergoing a second I&D increased by 9.6 %.

**Table 3:** Logistic regression analysis for key features at presentation

Multiple surgeries	Coefficient (*β*)	SE	*p*-Value	OR	95 % CI
Intercept	−4.545	0.346			
Positive blood culture	1.360	0.609	0.025*	3.9	0.167–2.553
CRP at presentation	0.096	0.035	0.005*	1.1	0.028–0.163

Logistic regression analysis of key clinical and diagnostic factors at the time of initial presentation in children with septic arthritis

*CRP* C-reactive protein; *SE* standard error; *OR* odds ratio; *CI* confidence interval

* p ≤ 0.05

Of the 21 patients who underwent multiple surgeries, 14 children (66.7 %) required two I&Ds, two children (9.5 %) required three, and one child (4.8 %) required four I&Ds. Three patients who presented critically ill underwent six, six, and eight surgeries, respectively, for eradication of infection; additionally, these patients required further surgery for delayed primary closure and/or suture removal under anesthesia. One final patient underwent two I&Ds during their index hospitalization, was later readmitted with recurrent septic arthritis in the same joint, and required an additional three surgeries. These children had aggressive organisms, developed septic arthritis in additional joints, and/or progressed to osteomyelitis or intra-muscular abscesses that were not apparent at initial presentation. The distribution of joints which required multiple surgeries included the hip (38.1 %), knee (28.6 %), ankle (4.8 %), and wrist (4.8 %). Two children (9.5 %) progressed to bilateral septic hips and one (4.8 %) progressed to bilateral septic knees when only one side was infected originally. One child (4.8 %) presented with a hip septic arthritis and then developed a contralateral hip septic trochanteric bursitis, and one child (4.8 %) who presented with bilateral septic hips required repeat surgery of both hips. The distribution of infected joints was similar between the multiple surgeries cohort and the entire study population; this study did not demonstrate a particular joint that was more prone to persistent infection. The median length of hospital stay for patients who required one or multiple surgeries was 6 days [interquartile range (IQR) = 3] and 13 days (IQR = 17), respectively (*z* = −6.15; *p* = 0.000). As advised by the infectious disease service, the total cumulative antibiotic course was a median of 4.25 weeks (IQR = 1.25) in the single surgery cohort, and 8.5 weeks (IQR = 5) in the multiple surgery cohort (*z* = −6.1; *p* = 0.000).

**Fig. 1:**
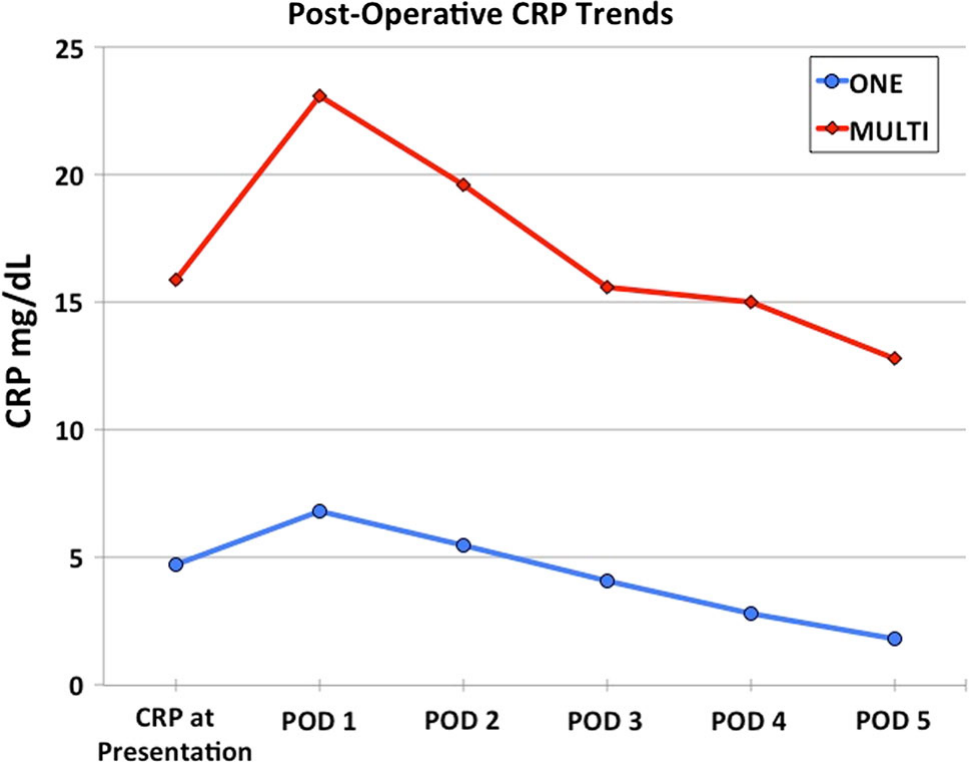
Post-operative C-reactive protein (CRP) trends in children who required a single (*ONE*) or multiple (*MULTI*) surgical debridements. Compared to children who clinically improved after only a single surgery, CRP at initial presentation was twice as high in children who failed a single surgical debridement (*p* = 0.005), and post-operatively, CRP remained nearly three-fold higher at all time points (all *p* = 0.000)

Post-operative CRP trends in children who required one or multiple operative interventions are presented in Table 4 and Fig. 1. We compared CRP values between groups using an ANOVA with repeated measures with a Greenhouse–Geisser correction. The mean scores for CRP concentration were statistically significantly different between time points [F(6, 340) = 12.7, *p* = 0.000)]. Post-hoc tests using Bonferroni correction revealed that the mean scores for CRP values differed significantly among all post-operative days (POD) 1–5 (*p* = 0.000) (Table 4).

**Table 4:** Post-operative trends in CRP

CRP (mg/dL)	Single surgery		Multiple surgeries		*p*-Value
	Mean (SD), *n* = 84	Median (IQR)	Mean (SD), *n* = 21	Median (IQR)	
POD 1	9.42 (±7.13)	6.80 (8.65)	23.32 (10)	23.1 (14)	0.000*
POD 2	8.10 (±6.54)	5.5 (7.4)	19.41 (±9.43)	19.6 (9.8)	0.000*
POD 3	6.04 (±5.54)	4.1 (4.7)	15.83 (±8.04)	15.6 (10.7)	0.000*
POD 4	4.31 (±4.27)	2.8 (4.05)	13.23 (±7.37)	15 (11.4)	0.000*
POD 5	3.14 (±3.37)	1.8 (2.5)	13.24 (±5.65)	12.8 (1.5)	0.000*

Post-operative C-reactive protein (CRP) trends in children with septic arthritis who required either a single or multiple debridements for resolution of infection. The values reported for the multiple surgery cohort represent CRP levels following the first surgery only

*SD* standard deviation; *IQR* interquartile range; *POD* post-operative day

* *p* ≤ 0.05

**Fig. 2:**
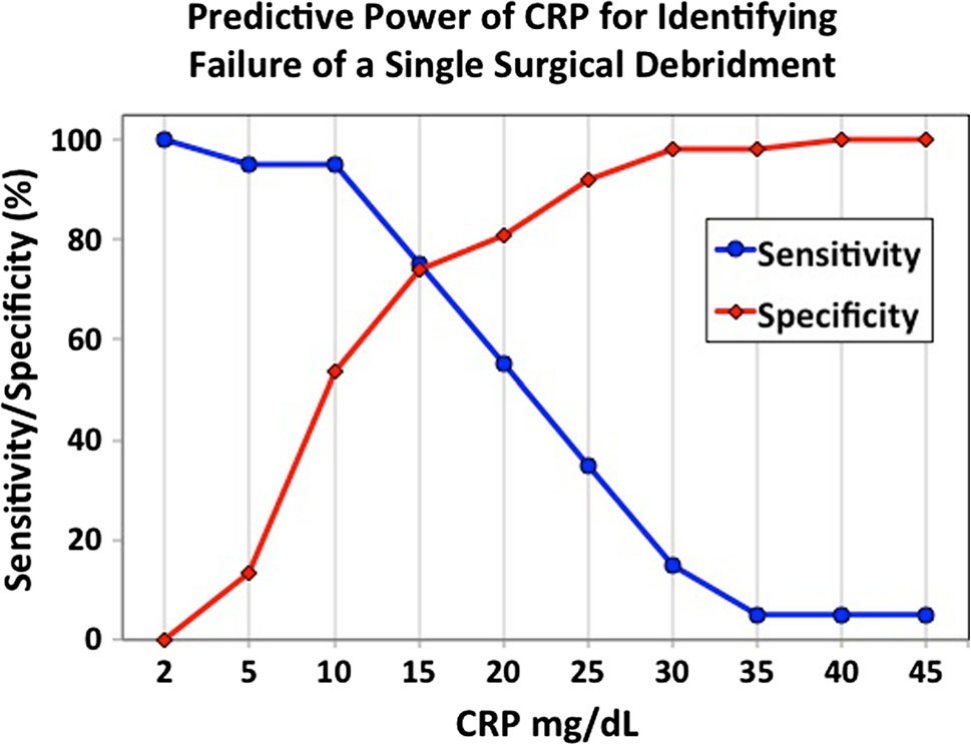
Sensitivity and specificity for failing a single debridement and requiring a second surgery at different cutoff values for C-reactive protein (*CRP*) at initial presentation. A CRP level of 15 mg/dL was found to be the optimal cut-point for predicting when a patient would undergo a second debridement

ROC curve analysis was performed to demonstrate the strength of the predictive model (see the supplementary material). The area under the curve was calculated at 0.799, which is considered almost excellent. Further, we calculated the sensitivities and specificities for failing a single surgery and requiring a revision surgery at different CRP cut-points (Fig. 2). An initial CRP level of 15 mg/dL was found to be the optimal cut-point for predicting that a patient would fail a single I&D and undergo a second surgery.

In the multiple surgeries cohort, there was a median of 4 days between the first and second surgeries, and by POD 5, 17 of 21 patients (80.9 %) had undergone a second procedure. In patients who required only one surgery, CRP declined to less than 2.0 mg/dL a median of 5 days post-operatively.

**Fig. 3:**
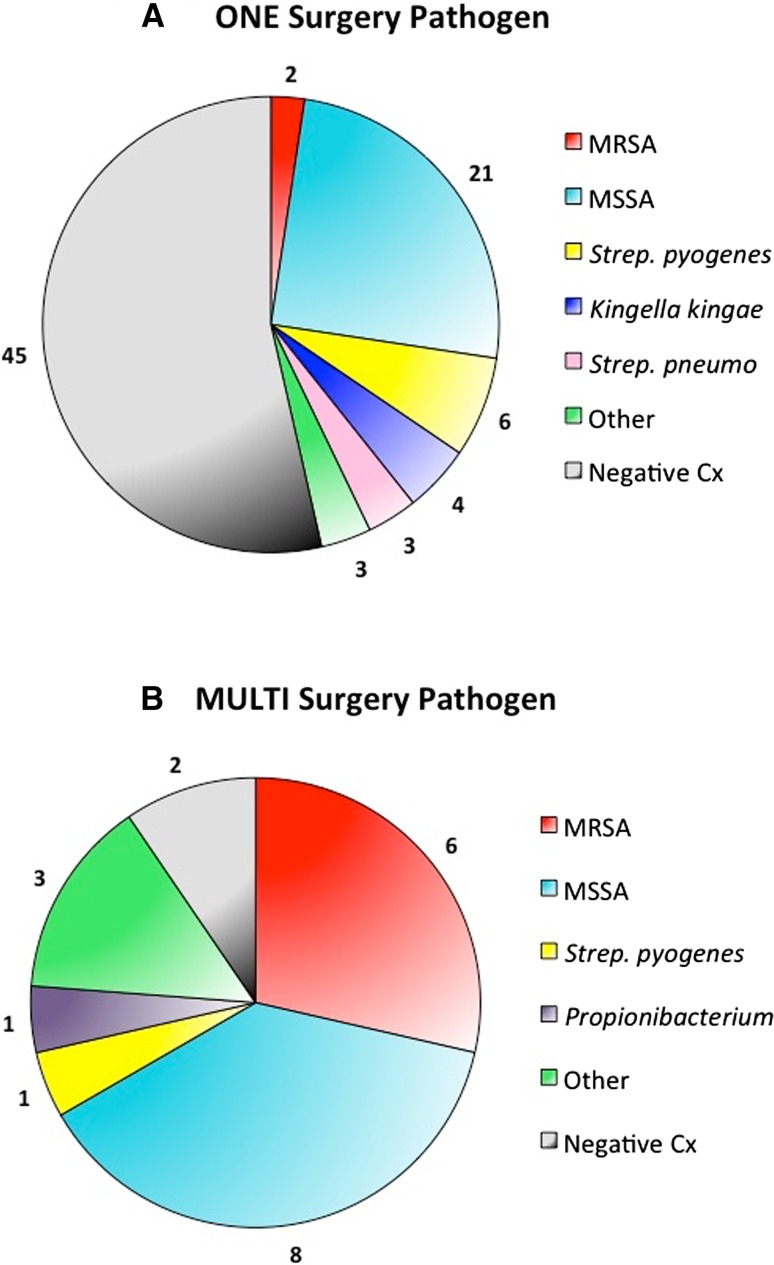
Causative bacterial species in pediatric septic arthritis. Combined joint and blood culture results in patients who required only a single (**a**) or multiple (**b**) debridements for eradication of infection

The rate of positive joint synovial fluid culture in the single and multiple surgery cohorts was 39.3 % (33 of 84 patients) and 76 % (16 of 21 patients), respectively. The rate of positive blood culture in the single and multiple surgery cohorts was 23.8 % (19 of 80 patients) and 66.7 % (14 of 21 patients), respectively; four patients from the single surgery cohort did not have blood cultures drawn. The rate of species identification from joint and/or blood culture in the single and multiple surgery cohorts was 36.2 % (38 patients) and 90.5 % (19 patients), respectively. Forty-eight patients (45.7 %) had negative cultures from all sources. The distribution of causative bacterial species isolated from either joint or blood cultures are shown in Fig. 3. There was an increased incidence of MRSA infection in children who required multiple surgeries (Fig. 3b).

## Discussion

The ability to prospectively identify children with septic arthritis who are likely to fail initial surgical management enables clinicians to more effectively manage these patients with closer surveillance, meticulous examination of the joint and more thorough excision (of non-viable infected tissue), earlier identification of continued infection, and initial family counseling. Furthermore, in community centers where urgent advanced imaging is not readily accessible, providers must rely heavily on patient history and routine laboratory parameters to make critical decisions regarding patient care. To our knowledge, this is the first study to report risk factors for failure of a single surgical intervention in pediatric septic arthritis on the basis of patient history and physical and routine laboratory studies. It is also the first to look at post-operative CRP trends comparing children who required one versus multiple surgeries for eradication of infection.

In the operative management of pediatric septic arthritis and osteomyelitis, CRP values peak in 1–3 days following surgery [14, 15, 19], and in uncomplicated cases, normalize within 7–12 days [14, 15, 19]. Persistently elevated or increasing CRP levels which do not follow the expected post-operative trend of peak and decline have been shown to be predictive of infection in adult populations [25] and complications in pediatric osteomyelitis [4, 9].

This study found that the CRP at initial presentation was twice as high in children who failed a single surgical intervention compared to those who clinically improved after a single surgery. An initial CRP of 5 mg/dL or less was highly sensitive and specific for requiring only one I&D, whereas an initial CRP of 30 mg/dL or greater was highly sensitive and specific for requiring multiple surgeries. Furthermore, in the multiple surgeries cohort, CRP remained nearly three-fold higher at all time points post-operatively. Both groups demonstrated a CRP peak on POD 1, which likely reflects both tissue trauma due to surgery [25, 26] and mechanical stimulation of the infectious nidi. However, in the multiple surgeries cohort, although the post-operative CRP levels generally trended down, presumably due to debulking of the infectious burden during the initial surgery and the action of intravenous antibiotics, they remained markedly elevated when compared to children who experienced clinical improvement after only a single surgery. Given that the differences in CRP levels between cohorts were statistically significant not only as early as POD 1 and 2, but also when the patient initially presented to the emergency department, these findings may help to prospectively identify at-risk children during a very early stage of their disease. Additionally, marked or persistent elevation of CRP values should alert the clinician to possible continued infection; this study found that a CRP cutoff of 15 mg/dL had the best sensitivity/specificity profile for predicting when a single surgery alone was likely to fail.

The present study found that several parameters widely considered mainstays in the diagnosis of pediatric musculoskeletal infection had little diagnostic or prognostic value in distinguishing patients who were more likely to fail a single surgical intervention. In accordance with prior studies in which ESR was unable to risk-stratify children with musculoskeletal infection [3, 15], we found no statistical difference in ESR at presentation between children who failed the initial surgery and those who did not. It may be that, in the setting of pediatric musculoskeletal infection, the greatest utility of ESR is in its negative predictive value [27]. Additionally, we found no significant difference in temperature at presentation, and, in fact, the median temperature did not exceed the typical cutoff of 38.5 °C in either cohort. Although WBC levels were ubiquitously elevated at presentation, in agreement with prior studies [14, 15, 27, 28], WBC was unable to distinguish between children who were likely to have an uncomplicated course and those who would require multiple surgeries. Additionally, the inability to bear weight on the affected extremity was omnipresent across the entire study population; while extremely helpful in heralding generalized musculoskeletal derangement [16, 17], it was of little prognostic value.

Bacteremia was also strongly predictive for failing a single surgical I&D with odds that were nearly four-fold greater in the presence of a positive blood culture. Additionally, the multiple surgeries cohort had a higher incidence of MRSA infection. When children with negative cultures were excluded, MRSA was the causative agent in only 5.1 % (2 of 39) of children who underwent a single surgery, but 31.6 % of children who required multiple surgeries (6 of 19). In addition to indicating a child is at increased risk for failing initial surgical management, positive blood cultures are sometimes the only way to identify the causative organism, given that the rate of species identification from synovial fluid can be as low as 30 % [21].

Predictably, the current study also found that children who failed a single surgical intervention remained, on average, hospitalized 7 days longer, and required four additional weeks of antibiotics, compared to children who improved after a single surgery. At the institution of the authors, not only does a prolonged clinical course pose challenges to the patient and their family, it also has wider implications for healthcare utilization and expenditure as well.

This study has several limitations. It is retrospective and, therefore, subject to information and selection bias. Furthermore, multiple surgeons participated in the care of these patients and there may have been some variability in their treatment during the perioperative period. Additionally, while many patients do undergo advanced imaging pre-operatively [26 % (27 children) with advanced imaging negative for osteomyelitis in this study and 20 patients excluded based on osteomyelitis found on MRI], it is not our institutional protocol to perform MRI on every child with septic arthritis, nor does every child with clinical failure undergo MRI prior to returning to the operating room. There are many advantages to MRI, namely the identification of osteomyelitis, soft tissue abscesses, or other concurrent pathology. However, like many healthcare settings nationwide, at the institution of the authors, it is neither temporally nor economically feasible to perform an urgent MRI on all children with suspected septic arthritis, nor do our providers believe it ubiquitously merits the risks of sedation in young children who are unable to lie still. Therefore, potentially, some children with a focus of osteomyelitis could have been falsely included. Also, in this investigation, the rate of negative species identification from either wound or blood cultures was 45.8 %. While high, this rate is consistent with previous studies of pediatric septic arthritis which report negative cultures in 30–66 % of patients [10, 20–22, 28]. Some species, such as *Kingella kingae*, are notoriously difficult to culture and have been identified via polymerase chain reaction (PCR) in up to 20.6 % of children with otherwise negative joint cultures [20]; these difficult-to-culture pathogens were likely the underlying pathogen in a cohort of children with otherwise negative cultures. However, a species was identified in 90.5 % of the multiple surgeries cohort, indicating that, if any children were falsely included, they likely came from the much larger single surgery cohort and would have only a minimal impact on the overall group behavior. Lastly, since immunocompromised children were excluded, we can make no conclusions regarding the risk of failed surgery in the patient population that is likely at the highest risk of all.

## Conclusions

This retrospective study demonstrated that a positive blood culture or marked elevation in CRP at presentation or on POD 1–4 were associated with revision surgery. The overall failure rate for a single surgical intervention was 20 %. These findings may help improve surgical planning for both the initial surgery in order to avoid revisions, as well as revision surgery should it be indicated.

## Electronic supplementary material

Below are the links to the electronic supplementary material.

Receiver operating characteristic (ROC) curve analysis to demonstrate the strength of the predictive model. The area under the curve was calculated at 0.799, which is considered almost excellent (TIFF 350 kb)

Single surgery cohort patient data (DOCX 120 kb)

Multiple surgery cohort patient data (DOCX 111 kb)
