# Supplementary figures and images for: Laboratory predictors for risk of revision surgery in pediatric septic arthritis
Source: J Child Orthop. 2016 May 12;10(3):247–54. doi: 10.1007/s11832-016-0736-6 (PMC4909651; doi:10.1007/s11832-016-0736-6)

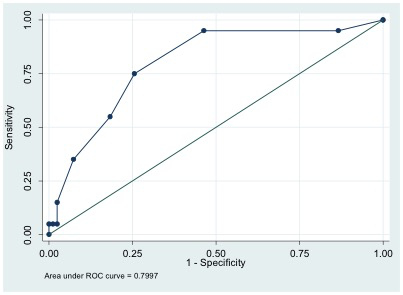

Supplement: Supplementary file 1 — Receiver operating characteristic (ROC) curve analysis to demonstrate the strength of the predictive model. The area under the curve was calculated at 0.799, which is considered almost excellent (TIFF 350 kb) [file 11832_2016_736_MOESM1_ESM.tiff]
