# Supplementary material for: Laboratory predictors for risk of revision surgery in pediatric septic arthritis
Source: J Child Orthop. 2016 May 12;10(3):247–54. doi: 10.1007/s11832-016-0736-6 (PMC4909651; doi:10.1007/s11832-016-0736-6)
Supplement: Supplementary file 2 — Single surgery cohort patient data (DOCX 120 kb) [file 11832_2016_736_MOESM2_ESM.docx]

| Supplement 2. Single Surgery Cohort Patient Data | | | | | | | | | |  |
| --- | --- | --- | --- | --- | --- | --- | --- | --- | --- | --- |
| Case | **Age (Years)** | **Joint at Presentation** | **PMH /**  **Active Medical Issues** | **Delay in Dx (Days)** | **Organism** | **WBC**  **(10^3^ cell/ mm^3^)** | **ESR (mm/ hr)** | **CRP (mg/dL)** | **Length of Stay (Days)** | |
| 1 | 0.6 | Hip | Active: URI 1.5 weeks prior | 10 | Negative Cx | 16.5 | 44 | 1.7 | 6 | |
| 2 | 3.3 | Knee | PMH: weight 10th percentile  Active: URI 1 week prior | 6 | Negative Cx | 11.8 | 43 | 1.8 | 8 | |
| 3 | 4.2 | Knee | Active: Lyme Disease | 1 | *Strep. viridans* | 9 | 23 | 3.1 | 7 | |
| 4 | 4.2 | Knee |  | 1 | MSSA | 11.7 | 20 | 4.2 | 5 | |
| 5 | 8.2 | Ankle |  | 3 | Negative Cx | 9.9 | 40 | 9.6 | 5 | |
| 6 | 12.7 | Hip |  | 8 | MRSA | N/A | N/A | 35.1 | 12 | |
| 7 | 5.4 | Hip | Active: URI | 3 | Negative Cx | 9.8 | 25 | 3 | 7 | |
| 8 | 14.9 | Hip | PMH: autism | 2 | Negative Cx | 15.2 | 11 | 7 | 6 | |
| 9 | 9.8 | Hip | PMH: beta-thalassemia trait  Active: post-streptococcus glomerulonephritis. | 1 | Negative Cx | 17.6 | 25 | 0.9 | 12 | |
| 10 | 6.9 | Hip | Active: otitis media 2 weeks prior | 3 | Negative Cx | 15.6 | 54 | 15.8 | 8 | |
| 11 | 12.1 | Elbow |  | 10 | MSSA | 8.6 | 38 | 8.9 | 8 | |
| 12 | 0.8 | Knee | Active: croup 2 weeks prior | 3 | *Strep. pneumoniae* | 16.8 | 86 | 6.7 | 6 | |
| 13 | 11.1 | Hip |  | 5 | Negative Cx | 10 | 38 | 4.2 | 7 | |
| 14 | 0.3 | Elbow |  | 4 | *Strep. pneumoniae* | 11.1 | 129 | 13.9 | 7 | |
| 15 | 6.7 | Hip | Active: otitis media 2 weeks prior | 5 | Negative Cx | 10.8 | 31 | 4.8 | 8 | |
| 16 | 1.6 | Hip |  | 4 | Negative Cx | 11.8 | 50 | 4.1 | 5 | |
| 17 | 1.0 | Ankle |  | 2 | Negative Cx | 17.1 | 37 | 6.2 | 4 | |
| 18 | 1.2 | Knee | PMH: absence seizures | NA | Negative Cx | 14.2 | 63 | 2.1 | 6 | |
| 19 | 6.6 | Hip | PMH: Tetralogy of Fallot, pulmonary artery stenosis s/p stents, ASD, VSD s/p bovine patch | 1 | Negative Cx | 9.7 | 7 | 8.7 | 5 | |
| 20 | 15.9 | Knee |  | 12 | Negative Cx | N/A | 95 | 30.2 | 10 | |
| 21 | 7.4 | Hip |  | 1 | Negative Cx | 12.8 | 87 | 13 | 4 | |
| 22 | 9.6 | Hip |  | 6 | Negative Cx | N/A | N/A | N/A | 7 | |
| 23 | 1.8 | Hip |  | 7 | Negative Cx | 27.6 | 106 | 21.1 | 8 | |
| 24 | 7.1 | Subtalar |  | 5 | Negative Cx | 16.9 | 95 | 6.6 | 5 | |
| 25 | 2.7 | Shoulder |  | 7 | Capnocytophaga species | 9.7 | 77 | 2.4 | 5 | |
| 26 | 1.3 | Knee |  | 8 | Negative Cx | 15.8 | 65 | 4.5 | 5 | |
| 27 | 4.3 | Knee |  | 1 | MSSA | 7.7 | 7 | 3.1 | 7 | |
| 28 | 5.7 | Hip |  | 7 | MSSA | 10 | 42 | 4.1 | 2 | |
| 29 | 1.1 | Hip |  | 4 | Negative Cx | 17 | 70 | 7 | 6 | |
| 30 | 8.8 | Hip | Active: excoriations from rash 1 week prior | 8 | Negative Cx | 12.7 | 71 | 3 | 5 | |
| 31 | 3.0 | Hip | PMH: GERD | 2 | MSSA | 7.8 | 26 | 22.5 | 10 | |
| 32 | 0.7 | Hip | Active: varicella zoster, skin excoriations | 3 | MSSA | 17 | 86 | 16.5 | 4 | |
| 33 | 9.3 | Hip | PMH: MRSA STI 1 year prior | 21 | Negative Cx | 9.2 | 30 | 1.7 | 6 | |
| 34 | 6.9 | Hip |  | 0 | Negative Cx | 14.2 | 20 | 0.8 | 6 | |
| 35 | 4.7 | Hip | PMH: autism  Active: *E. coli* UTI. Sinusitis 2 weeks prior | 1 | Negative Cx | 10.8 | 9 | 3.2 | 3 | |
| 36 | 0.8 | Elbow |  | 1 | MSSA | 16.5 | 20 | 3.5 | 5 | |
| 37 | 3.3 | Knee |  | 5 | Negative Cx | 13.9 | 46 | 2.6 | 7 | |
| 38 | 6.3 | Hip | Active: streptococcal-related rash 1.5 weeks prior | 7 | *Strep. pyogenes* | 24.9 | 80 | 20 | 8 | |
| 39 | 7.6 | Ankle | PMH: eczema | NA | MSSA | 13.2 | 89 | 7.3 | 6 | |
| 40 | 7.8 | Elbow | Active: otitis media 2 weeks prior | 2 | Negative Cx | 8.8 | 30 | 13.8 | 6 | |
| 41 | 0.3 | Hip | Active: *E. coli* UTI | 4 | MSSA | 8.1 | 34 | 2.2 | 4 | |
| 42 | 2.9 | Knee |  | 2 | Negative Cx | 16.9 | 57 | 1.8 | 7 | |
| 43 | 1.4 | Elbow |  | 3 | *Strep. pyogenes* | 13.3 | 81 | 17.8 | 9 | |
| 44 | 1.9 | Knee | Active: URI 2 weeks prior | 5 | Negative Cx | 11.8 | 40 | 3.3 | 6 | |
| 45 | 8.8 | Knee | PMH: remote MRSA STI | 5 | MRSA | 12.2 | 80 | 17.1 | 17 | |
| 46 | 3.1 | Hip |  | 1 | Negative Cx | 16.3 | 41 | 7.1 | 5 | |
| 47 | 8.4 | Hip | Active: empyema 4 weeks prior | 1 | Negative Cx | 9.2 | 23 | 3.4 | 6 | |
| 48 | 1.6 | Knee |  | 5 | Negative Cx | 17.7 | 27 | 1.2 | 4 | |
| 49 | 7.5 | Hip | PMH: MHE | 2 | MSSA | 9.3 | 31 | 8.7 | 9 | |
| 50 | 1.8 | Hip |  | 2 | MSSA | 11.3 | 28 | 19.1 | 12 | |
| 51 | 2.3 | Knee | PMH: short gut syndrome, TPN dependent. Prior MSSA & enterobactor infections | 2 | MSSA | 2.6 | 23 | 7.2 | 11 | |
| 52 | 1.1 | Shoulder |  | 14 | *Kingella kingae* | 19.1 | 80 | 2.8 | 5 | |
| 53 | 2.1 | Hip | Active: otitis media 2 weeks prior | 2 | Negative Cx | 21.9 | 42 | 2.2 | 6 | |
| 54 | 6.5 | Hip |  | 2 | Negative Cx | 16.2 | 43 | 6.8 | 5 | |
| 55 | 6.4 | Ankle |  | 0 | MSSA | 9.2 | 18 | 2 | 4 | |
| 56 | 3.8 | Knee | PMH: gastroschesis, short gut syndrome, chronic paronychia | 11 | Negative Cx | 6.2 | 39 | 3.7 | 7 | |
| 57 | 11.7 | Knee | PMH: ADHD, precocious puberty | 7 | Negative Cx | 9 | 38 | 4.1 | 7 | |
| 58 | 1.6 | Ankle |  | 4 | Negative Cx | 14.6 | 50 | 1.7 | 2 | |
| 59 | 7.9 | Hip | PMH: ADHD | 1 | Negative Cx | 23.5 | 10 | 3.5 | 5 | |
| 60 | 1.5 | Ankle |  | 5 | *Kingella kingae* | 15.2 | 32 | 3.6 | 4 | |
| 61 | 9.0 | Hip | Active: streptococcal pharyngitis 3 weeks prior | 1 | Negative Cx | 15.4 | 7 | 1.2 | 7 | |
| 62 | 1.8 | Knee |  | 1 | *Strep. pyogenes* | 13.5 | 102 | 19.6 | 9 | |
| 63 | 9.7 | Hip |  | 2 | *Kingella kingae* | 13.9 | 34 | 16.1 | 12 | |
| 64 | 0.3 | Ankle | PMH: eczema, recurrent otitis media | 4 | Negative Cx | 12.1 | 60 | 7.4 | 5 | |
| 65 | 5.9 | Hip | Active: streptococcal pharyngitis | 0 | Negative Cx | 17 | 20 | N/A | 3 | |
| 66 | 14.7 | Hip & shoulder | PMH: ADHD, OCD, Asperger’s  Active: sinusitis | 3 | *Strep. pyogenes* | 6.3 | 12 | 22.3 | 16 | |
| 67 | 1.2 | Knee | Active: URI | 4 | *Strep. pyogenes* | 19.2 | 96 | 5.6 | 6 | |
| 68 | 1.6 | Knee | Active: URI | 3 | Negative Cx | 12 | 29 | 3.2 | 4 | |
| 69 | 1.2 | Hip |  | 1 | Negative Cx | 13.5 | 38 | 4.7 | 5 | |
| 70 | 7.2 | Hip |  | 6 | Negative Cx | 7.5 | 78 | 13 | 8 | |
| 71 | 12.6 | Knee |  | 4 | MSSA | 5 | 44 | 7 | 6 | |
| 72 | 2.0 | Knee | Active: URI | 0 | Negative Cx | 9.1 | 30 | 4.1 | 6 | |
| 73 | 7.9 | Knee |  | 1 | Negative Cx | 11.1 | 28 | 4.7 | 8 | |
| 74 | 3.9 | Knee | Active: superficial laceration & cellulitis 1 week prior | 1 | MSSA | 10.8 | 54 | 3.7 | 8 | |
| 75 | 0.5 | Hip |  | 2 | MSSA | 12.9 | 36 | 4.4 | 8 | |
| 76 | 2.6 | Knee |  | 1 | MSSA | 10.6 | 12 | 3.3 | 7 | |
| 77 | 0.8 | Hip |  | 1 | MSSA | 15.6 | 18 | 10.6 | 10 | |
| 78 | 12.3 | Knee | Active: superficial laceration & cellulitis 1.5 weeks prior | 0 | *Serratia marcescens* | 9.5 | 111 | 8 | 6 | |
| 79 | 1.4 | Shoulder |  | 8 | *Kingella kingae* | 7.6 | 53 | 1.5 | 6 | |
| 80 | 0.5 | Hip |  | 1 | MSSA | 21.5 | 28 | 12.7 | 8 | |
| 81 | 5.7 | Knee | PMH: bicuspid aortic valve, aortic stenosis & insufficiency  Active: endocarditis, streptococcal pharyngitis | 7 | MSSA | 7.7 | N/A | 20.7 | 12 | |
| 82 | 11.7 | Hip | Active: *C. difficile* colitis | 2 | MSSA | 11.6 | 52 | 16.4 | 15 | |
| 83 | 7.2 | Hip | Active: PNA | 14 | *Strep. pyogenes* | 10.8 | 109 | 3.4 | 15 | |
| 84 | 3.2 | Ankle | Active: otitis media | 6 | *Strep. pneumoniae* | 16.3 | 101 | 6.9 | 6 | |
| Single surgery cohort patient data. All patients underwent a single surgery, and had an uneventful post-operative course. ADHD, attention deficit hyperactive disorder; OCD, obsessive compulsive disorder; UTI, urinary tract infection; URI, upper respiratory infection; MRSA, methicillin resistant *Staph. aureus*; MSSA, methicillin sensitive *Staph. aureus*; Cx, culture; WBC, white blood cell; ESR, erythrocyte sedimentation rate; CRP, C-reactive protein. | | | | | | | | | |  |
