# Supplementary material for: Laboratory predictors for risk of revision surgery in pediatric septic arthritis
Source: J Child Orthop. 2016 May 12;10(3):247–54. doi: 10.1007/s11832-016-0736-6 (PMC4909651; doi:10.1007/s11832-016-0736-6)
Supplement: Supplementary file 3 — Multiple surgery cohort patient data (DOCX 111 kb) [file 11832_2016_736_MOESM3_ESM.docx]

| Supplement 3. Multiple Surgery Cohort Patient Data | | | | | | | | | | | |
| --- | --- | --- | --- | --- | --- | --- | --- | --- | --- | --- | --- |
| Case | **Age (Years)** | **Joint at Presentation** | **PMH /**  **Active Medical Issues** | **Hospital Course** | **Surgery** | **Delay in Dx (Days)** | **Organism** | **WBC**  **(10^3^ cell/ mm^3^)** | **ESR (mm/ hr)** | **CRP (mg/dL)** | **Length of Stay (Days)** |
| 85 | 2.3 | Hip |  | Developed intramuscular thigh abscess 2 days after 1^st^ surgery, MRI showed OM, no recurrent hip effusion | I&D x2 | 4 | MSSA | 19.2 | 50 | 14.9 | 10 |
| 86 | 5.1 | Hip |  | Persistent hip effusion, elevated CRP, lack of clinical improvement 4 days after 1st surgery | I&D x2 | 2 | MSSA | 5.6 | 31 | 6.3 | 9 |
| 87 | 7.2 | Knee |  | ICU admission. Initial concern for NSTI (neg). Presented with isolated right knee SA, MRI negative for OM. Developed left knee SA & bilateral distal femur OM 2 days after 2^nd^ right knee surgery | I&D x6  Delayed wound closure x2 | 6 | MRSA | 7.3 | 29 | 15.9 | 41 |
| 88 | 6.5 | Hip |  | Presented with left hip SA, developed right hip SA 4 days after 1^st^ surgery | I&D x2, left hip twice, right once | 2 | Negative Cx | 19.2 | 68 | 13.7 | 10 |
| 89 | 1.3 | Hip |  | Persistent hip effusion, increasing CRP and lack of clinical improvement 3 days after 1st surgery  Re-admitted 2 weeks after discharge with recurrent hip effusion and required 3 more I&Ds. No OM on MRI or on bone drilling | I&D x2, 1^st^ admission  I&D x3 2^nd^ admission | 10 | MSSA | 17 | 88 | 14.1 | 10 |
| 90 | 9.5 | Hip |  | Persistent hip effusion on post-op MRI (no OM), elevated CRP and lack of clinical improvement 3 days after 1st surgery | I&D x2 | 6 | MSSA | N/A | N/A | 15.8 | 10 |
| 91 | 4.8 | Hip |  | Presented with left hip SA, CT negative for OM/abscess. Developed right hip extra-articular abscess and multifocal pyomyositis 3 days after 1^st^ surgery, MRI negative for OM | I&D x2 | 4 | MRSA | 3.9 | 60 | 27.9 | 14 |
| 92 | 5.7 | Hip |  | Lack of clinical improvement, elevated CRP 3 days after first surgery. Post-op MRI showed possible OM/abscess; small effusion, no OM/abscess found at 2^nd^ surgery | I&D x2 | 2 | MSSA | 11.7 | 102 | 22 | 10 |
| 93 | 8.1 | Hip | Active: Superficial abrasion 5 days prior | Initially right hip SA, no OM on bone drilling in OR. Developed right femur OM and left hip SA 3 days after 1^st^ surgery | I&D x6  Dressing change under ANES x3  Delayed wound closure x1 | 2 | MRSA | 21.1 | 25 | 14.9 | 27 |
| 94 | 4.9 | Hip |  | Presented with right hip SA, MRI negative for OM. Lack of clinical improvement, increasing CRP and purulent wound drainage after 1^st^ and 2^nd^ surgeries. MRI prior to 3^rd^ surgery suggested OM but not found on bone drilling | I&D x3 | 3 | MSSA | 13 | 51 | 7.9 | 13 |
| 95 | 15.1 | Hip |  | Lack of clinical improvement and elevated CRP 9 days after 1^st^ OR. MRI after 1^st^ OR suggested deep pelvic abscess and OM but not found at 2nd surgery | I&D x2 | 2 | MSSA | 14.8 | 77 | 24.9 | 21 |
| 96 | 1.6 | Knee |  | Persistent purulent drainage, recurrent effusion, elevated CRP 4 days after 1^st^ surgery | I&D x2 | 8 | Negative Cx | 22 | 20 | 1.1 | 11 |
| 97 | 14.4 | Knee | Active: Intra-articular foreign body s/p assault 2 weeks prior | Clinical deterioration, recurrent effusion, elevated CRP 4 days after 1^st^ surgery | I&D x2 | 15 | *Propioni-bacterium* | N/A | N/A | N/A | 17 |
| 98 | 14.0 | Knee | PMH: Migraines  Active: infected ingrown toenail | Clinical deterioration, elevated CRP and OM 1.5 weeks after 1^st^ OR. Lack of clinical improvement and elevated CRP after 2^nd^ surgery. No purulence on bone drilling | I&D x3 | 14 | MRSA | 17.5 | 78 | 29.9 | 38 |
| 99 | 6.3 | Knee | Active: URI | Clinical deterioration, increasing CRP, recurrent effusion 5 days after 1^st^ surgery. MRI suggested possible OM, no purulence in canal on bone drilling | I&D x2 | 4 | MSSA | 7.3 | 18 | 6.2 | 11 |
| 100 | 11.4 | Knee | PMH: Asthma | Increasing pain and CRP, and purulent wound drainage 2 days after 1^st^ surgery. MRI demonstrated recurrent effusion and possible OM | I&D x2 | 0 | *Fusobacterium* | 6.9 | 80 | 8.2 | 11 |
| 101 | 10.9 | Knee |  | Developed increasing pain and CRP 4 days after 1^st^ surgery. Developed proximal tibial OM | I&D x2 | 10 | *Strep. anginosus* | 23.2 | 104 | 24.6 | 11 |
| 102 | 7.4 | Ankle | Active: MRSA cellulitis, Influenza A URI, C. difficile colitis, GI bleed | ICU admission. Initial concern for NSTI (negative) and compartmental syndrome; anterior and lateral compartments opened with equivocal pressure measurements. Recurrent ankle effusion, elevated CRP 2 days after 1^st^ surgery. Developed subperiosteal abscess, but not OM, after 2^nd^ surgery | I&D x4 | 7 | MRSA | 6.6 | 40 | 18.9 | 20 |
| 103 | 17.5 | Wrist | Active: Lemierre's Syndrome, intracranial & trapezius abscesses, internal jugular & transverse sinus thrombosis | ICU admission. Continued wrist pain, elevated CRP 4 days after initial surgery. Developed volar wrist soft tissue abscess, demonstrated on MRI | I&D x2 | 10 | *Fusobacterium necrophorum & Strep. anginosus* | 23.6 | 11 | 23.7 | 27 |
| 104 | 11.3 | Hip | PMH: ADHD  Active: infected pierced ear 2 weeks prior. Septic shock, pericardial effusion, superficial femoral vein occlusive thrombus, septic renal & pulmonary emboli | ICU admission. Presented with hip SA, MRI negative for OM. Core decompression performed at 1^st^ surgery with no OM. Developed right femur draining wound and OM 2 days after 1^st^ surgery. Developed left hip SA after 2^nd^ right hip surgery.  Re-admitted 6 weeks after DC for superficial right hip abscess debridement | I&D x8  Dressing change under ANES x2  Plastic surgery closure x1 | 5 | MRSA | 4.9 | 51 | 43.4 | 80 |
| 105 | 6.3 | Bilateral Hips | Active: septic shock, pancreatitis, colitis, hepatitis, septic pulmonary emboli, plural effusion, coronary artery dilatation | ICU admission. Presented with bilateral hip SA, CT negative for OM. Developed recurrent hip effusions and soft tissue abscesses with necrotic muscle bilaterally 12 days after 1^st^ surgery. Developed right knee SA 3.5 weeks after 1^st^ surgery | I&D x6  Suture removal under ANES x1 | 5 | *Strep. pyogenes* | 4.7 | 8 | 18.4 | 70 |
| Multiple surgery cohort patient data. All patients underwent a multiple surgeries for resolution of infection, and post-operative course notable for the issues listed. ADHD, attention deficit hyperactive disorder; ANES, anesthesia; CT, computed tomography; CRP, C-reactive protein; Cx, culture; ESR, erythrocyte sedimentation rate; GI, gastrointestinal; I&D, incision and drainage; MRI, magnetic resonance imaging; MRSA, methicillin resistant *Staph. aureus*; MSSA, methicillin sensitive *Staph. aureus*; NSTI, necrotizing soft tissue infection; OM, osteomyelitis; SA, septic arthritis; URI, upper respiratory infection; UTI, urinary tract infection; WBC, white blood cell | | | | | | | | | | | |
